# Vivid illusions and realtime feedback in VR-improved motor imagery and motivation of stroke patients with impaired motor imagery

**DOI:** 10.3389/fneur.2025.1629587

**Published:** 2025-11-07

**Authors:** Yanqing Xiao, Yang Gao, Hongming Bai, Ning Li, Xiao E. Cai, Jia-Sheng Rao, Aimin Hao, Xiaoguang Li, Jia Zheng

**Affiliations:** 1 Beijing Key Laboratory for Biomaterials and Neural Regeneration, Beijing Advanced Innovation Center for Biomedical Engineering, School of Biological Science and Medical Engineering, Beihang University, Beijing, China; 2 The State Key Laboratory of Virtual Reality Technology and Systems, Beihang University, Beijing, China; 3 Research Unit of Virtual Body and Virtual Surgery (2019RU004), Chinese Academy of Medical Sciences, Beijing, China; 4 Beijing Mentougou District Hospital, Beijing, China; 5 Department of Rehabilitation Medicine, Beijing Haidian Hospital, Beijing, China; 6 Beijing Children's Hospital, Capital Medical University, Beijing, China

**Keywords:** stroke, motor imagery, virtual reality, sense of embodiment, motivation

## Abstract

Virtual reality (VR) enhances subjective imagery experience. However, no previous studies have investigated whether VR can improve subjective imagery experience in stroke patients who specifically present with motor imagery (MI) impairment. The present work aimed to examine the effect of interactive virtual reality on subjective imagery experience in such a patient population. Twenty-eight stroke patients with hemiplegia who were specifically recruited based on objectively assessed motor imagery impairment (as measured by the KVIQ-10) participated in the study. Following interactive VR training, all subjects completed questionnaires assessing subjective imagery experience, sense of embodiment, motivation, and adverse reactions. A 1-week follow-up was conducted. The results showed that these patients with pre-existing MI impairment did not differ from the healthy control group in terms of sense of embodiment or subjective imagery experience under VR conditions. Furthermore, the patient group demonstrated significantly higher system acceptance in motivation assessments compared to the healthy controls. Most patients voluntarily recalled the VR scenes during the follow-up week, whereas participants in the control group did not. These findings indicate that stroke patients with overt motor imagery deficits can benefit from the proposed VR rehabilitation system, supporting its potential for further development in VR-based neurorehabilitation programs.

## 1 Introduction

Stroke represents a major cause of disability and mortality worldwide (1). Early and intensive active rehabilitation therapy is crucial for maximizing motor function restoration in stroke patients (2, 3). Motor imagery (MI) refers to the ability to mentally execute a movement without moving the body (4). MI in rehabilitation helps enhance the reorganization of damaged brain regions via recruitment of undamaged neurons and brain activity enhancement in other neuronal networks (5), making it a potential method for early active rehabilitation training.

In recent years, the application of MI has expanded beyond standalone use, increasingly integrating with various neuromodulation and interface technologies to form multimodal, closed-loop combined intervention strategies, significantly enhancing rehabilitation outcomes. Closed-loop systems based on brain-computer interfaces (BCI) decode MI-related electroencephalographic signals (such as μ/β rhythm suppression) in real-time and translate them into multimodal sensory feedback (6) (1) or drive peripheral devices (e.g., functional electrical stimulation, FES) (7), thereby establishing a reinforced “imagery-feedback-execution” loop. This integrated BCI-FES approach significantly enhances the coupling between motor intention and actual movement output, promoting the remodeling of motor pathways. On the other hand, non-invasive brain stimulation techniques—such as transcranial direct current stimulation (tDCS) and repetitive transcranial magnetic stimulation (rTMS)—can be used synergistically with MI. By modulating cortical excitability (e.g., tDCS increasing excitability in the affected hemisphere, rTMS rebalancing interhemispheric inhibition), they create a more favorable neural environment for MI, thereby more effectively inducing activation and functional reorganization in the affected brain regions (8, 9).

It is noteworthy that the effectiveness of these integrated advanced technologies highly depends on a core factor: the patient's motor imagery ability. Significant individual differences exist in aspects such as imagery vividness and cortical activation patterns (e.g., the degree of sensorimotor rhythm lateralization), which directly impact the decoding efficiency of BCI systems and the quality of closed-loop feedback (10, 11). Consequently, current personalized rehabilitation paradigms emphasize the assessment of patients' imagery ability prior to intervention and may incorporate neurofeedback mechanisms during training to gradually guide patients in learning how to effectively modulate their own brain activity, thereby maximizing the benefits of combined therapies.

However, patients with severe motor paralysis (e.g., after stroke or spinal cord injury) face significant neural and clinical challenges when performing MI. At the neural mechanism level, patients often exhibit markedly reduced or absent activation in key motor control brain regions (such as the primary motor cortex and sensorimotor cortex), making it difficult for them to generate effective MI-related brain activity patterns similar to those in healthy individuals (12, 13). Furthermore, the specific location and extent of paralysis lead to diverse patterns of brain functional reorganization, further increasing inter-individual heterogeneity in neural activity (12). In clinical applications, despite the rehabilitative potential of MI-BCI technology, systems often struggle to reliably recognize motor intentions due to issues such as low EEG signal strength, poor signal-to-noise ratio, and high non-stationarity in patients (14, 15). Particularly noteworthy is that some patients, due to insufficient activation in key regions such as the frontoparietal attention network and supplementary motor area (SMA), are unable to effectively control BCI systems (11, 16).

To address these difficulties, current research is dedicated to developing multimodal feedback strategies (e.g., integrating visual, tactile, and proprioceptive stimuli) (6) to enhance neural plasticity and regional brain activation during MI (8). For instance, the use of mirror video feedback from the non-paralyzed limb has been shown to improve the vividness and execution quality of motor imagery, thereby indirectly promoting functional recovery of the paralyzed limb (17).

Virtual Reality (VR) technology is rapidly gaining traction in the field of neuromotor rehabilitation, offering innovative and effective interventions for functional recovery following central nervous system injuries. By creating immersive, multi-sensory simulated environments, VR systems provide highly controllable rehabilitation conditions that integrate visual, auditory, and tactile feedback to enhance patient engagement and motivation, thereby promoting motor learning and neuroplasticity (18, 19). Current VR applications in rehabilitation have evolved from simple non-immersive desktop platforms to fully immersive systems incorporating head-mounted displays (HMDs), motion capture, and force feedback technology. These advanced systems more effectively induce a Sense of Embodiment (SOE)—comprising self-location, agency, and body ownership (20)—which serves as a key mechanism for improving rehabilitation outcomes.

Recent research emphasizes the critical importance of ecological validity and feedback design in VR environments for rehabilitation efficacy. Modern VR-based rehabilitation systems can simulate functional everyday tasks and provide real-time, multimodal feedback adjusted to patient performance, thereby optimizing motor output and activating central neural pathways (21, 22). For instance, through real-time motion tracking, patient movements can be accurately captured and mapped onto virtual avatars, allowing individuals to observe their own motions from first- or third-person perspectives. This enhances the congruence between motor intention and visual feedback and improves the quality and execution of MI (18, 19, 23–25). Furthermore, evidence suggests that gamified elements and adaptive difficulty mechanisms in VR environments significantly increase patient participation and treatment adherence, which is particularly valuable for populations with neurological impairments such as stroke and spinal cord injury (26–28).

VR demonstrates unique advantages in motor imagery training. By providing rich visual illusions and real-time movement visualization, it assists patients—especially those with impaired MI ability, such as in the early stages of stroke—in better performing mental simulation of movements (19, 29). Moreover, VR training protocols integrated with neuromodulation strategies (e.g., closed-loop brain-computer interface feedback) can further regulate sensorimotor rhythms and balance cortical excitability, thereby accelerating the remodeling of motor pathways (30, 31). Of note, recent systematic reviews indicate that VR-assisted training yields significant improvements in upper limb function, balance, and gait rehabilitation compared to conventional therapy, particularly when intervention dosage is adequate (32–35).

Despite these promising results, most current studies have focused on healthy individuals or chronic-phase patients, with relatively limited high-quality clinical trials targeting acute-phase populations or those with significant MI deficits. Moreover, the personalization of VR rehabilitation systems and the optimization of intervention parameters—such as immersion level, feedback type, and training intensity—require further investigation (22, 36).

This study aimed to evaluate a novel MI training system integrating fully immersive virtual reality with multimodal feedback, driven by sEMG signals from the unaffected wrist to control virtual ankle movement. We compared perceptual embodiment and MI performance between post-stroke patients with motor imagery deficits and healthy participants under conditions of immersive spatial representation and interactive feedback. The system's effect on patient motivation was also assessed. The findings demonstrate that the proposed approach is feasible and effective for rehabilitating stroke patients with MI impairments, offering a promising pathway for developing individualized and precise neurorehabilitation strategies tailored specifically to this population.

## 2 Material and methods

### 2.1 Participants

Totally 28 stroke cases (cerebrovascular accident [CVA] group) and 28 gender- and age-matched healthy individuals (control [CTL] group) assessed by physiotherapists and occupational therapists based on the Kinesthetic and Visual Imagery Questionnaire (KVIQ-10) (37) were included (Table 1). The study had approval from the Biomedical Ethics Committee of Beihang University (number BM20230165), and each participant provided signed informed consent.

**Table 1:** Characteristics of patients and healthy subjects.

**Characteristic**	**CVA (*n =* 28)**	**CTL (*n =* 28)**
**Age (** ***y*** **)**		
Mean	76.393	76.750
SD	7.385	4.873
Range	60–89	68–85
**Gender (** ***n*** **)**		
Male	15	14
Female	13	14
**Stroke course (** ***d*** **)**		NA
Mean	20.607	
SD	11.921	
Range	6–51	
**MRC**		NA
Range	0–2	
**Brunnstrom**		NA
Range	1–2	

CTL, control group; CVA, cerebrovascular accident group; MRC, Medical Research Council; NA, Not Applicable; SD, standard deviation.

All patients met the following inclusion criteria:

a) Mostly motor symptoms, with unilateral hemiplegia monitored by routine neurological assessment.
b) All cases were in stage 1–2 of the Brunnstrom Assessment scale.
c) Residual movement ability of the ankle of the affected side, assessed by the Medical Research Council (MRC) index ranging from 0 to 2, from no visible contraction to active movement but not against gravity.
d) Stroke course ranging between 3 days and 3 months.
e) Score of any item in the KVIQ-10 equal to or less than 2 (see Table 2).
f) Adequate cooperation and cognitive function to perform various activities, as assessed by the recruiting investigator.

**Table 2:** Rating subscales of the KVIQ-10.

**Visual (Image clarity)**	**Kinesthetic**
5: As clear as seeing	5: As intense as executing the action
4: Clear	4: Intense
3: Moderately clear	3: Moderately intense
2: Blurred	2: Mildly intense
1: No image	1: No sensation

Exclusion criteria were:

a) Serious cognitive impairment (score < 20 in the Mini Mental State Examination [MMSE]).
b) Serious ideomotor apraxia.
c) Serious language deficits, detected by clinical assessment.

In the CTL group, the participant with the score of any item in the KVIQ-10 assessment less than 3 was excluded.

### 2.2 Experimental procedure

**Figure 1:**
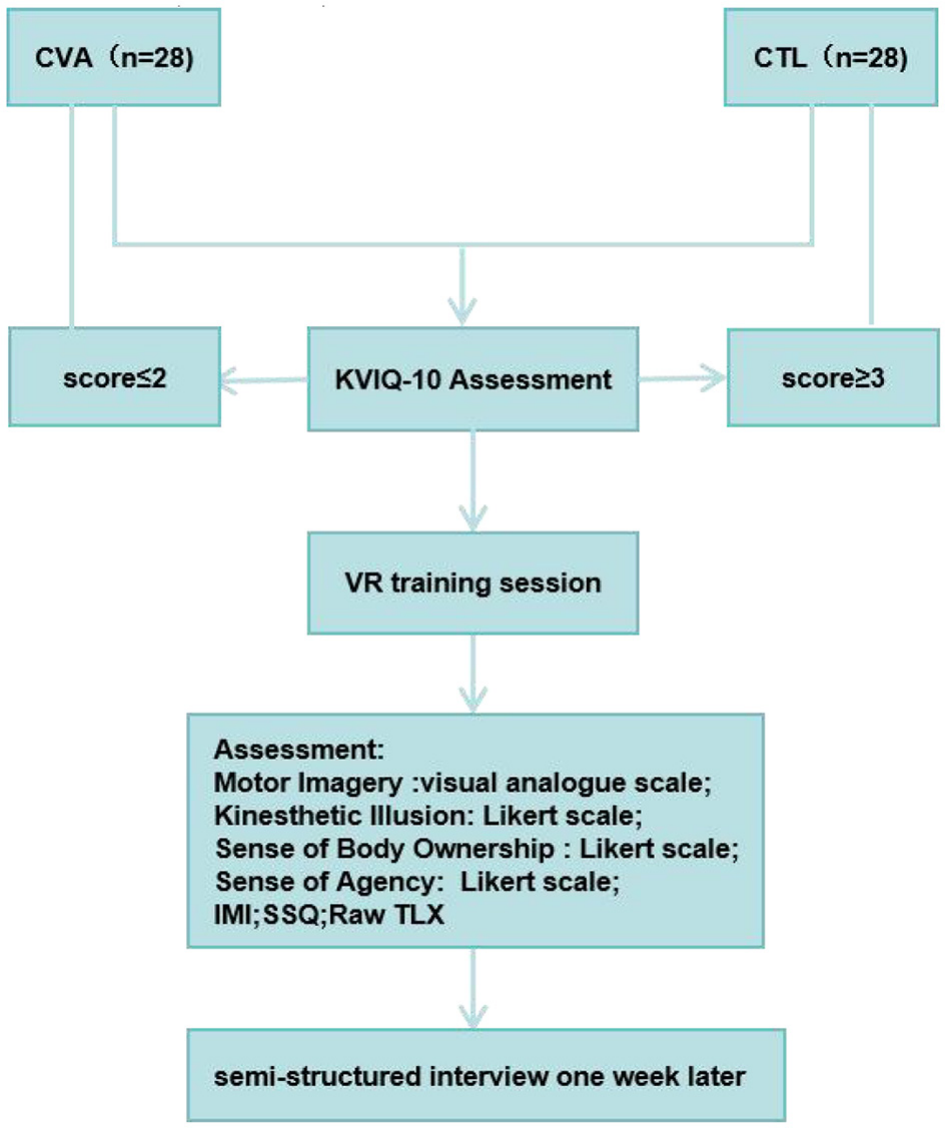
Experiment flowchart.

All participants were positioned in a supine inclined position and provided a Myo armband on the unaffected arm and wore the HTC Vive HMD. In individuals with thin arms, Myo Sizing Clips (https://www.thingiverse.com/thing:04751471) were printed to secure the contact. The VR training session lasted for 5 mins. For fatigue prevention, a 10-s rest was allowed after each minute of training. Patients were instructed to carry out dorsiflexion imagery of the affected ankle in the virtual scene at an enjoyable pace while carrying out wrist dorsiflexion of the unaffected one based on the imagined ankle's moving speed, focusing on sensations resulting from ankle's motor imagery. The motion of the virtual avatar was controlled by the sEMG of the contralateral unaffected wrist. In the healthy individuals control group, either the left wrist or the right wrist is randomly used to control the contralateral virtual ankle. After the training session, participants rated the perceived motor imagery vividness, motor imagery effort, kinesthetic illusion, sense of body ownership, sense of agency and motor control (38), the raw task load index (Raw TLX) (39, 40), and intrinsic motivation inventor (IMI) (41). Pre- and post-training, the simulator sickness questionnaire (SSQ) was administered (42). Following the training, paper questionnaires were immediately filled by each patient on site. In addition, all participants were interviewed 1 week after the experiment, where they were asked: “Did you often recall the VR video days ago during this week and try to practice it?” (see Figure 1).

### 2.3 VR training

**Figure 2:**
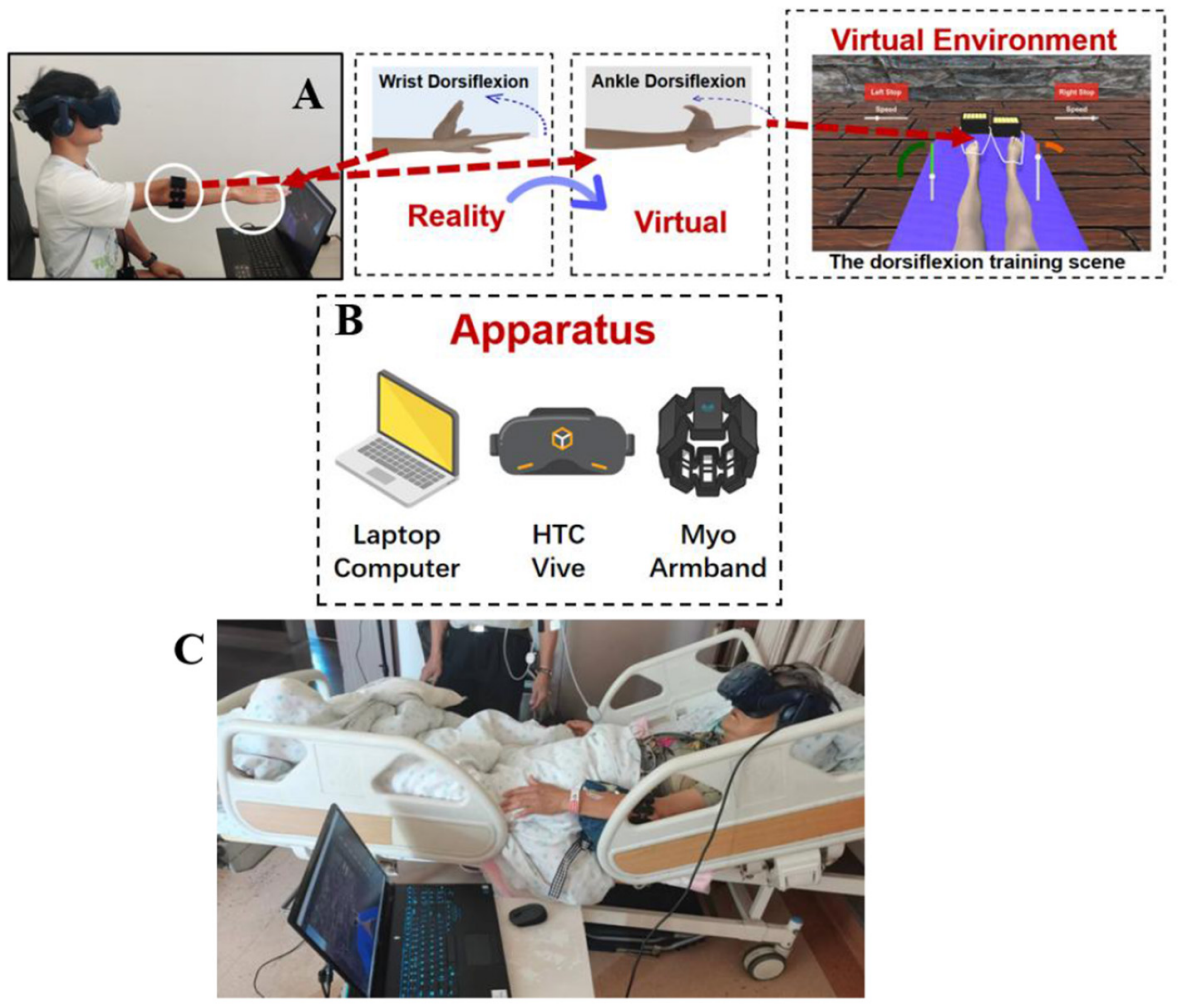
Schematic diagram of the virtual reality rehabilitation training system and its clinical application scenario. **(A)** Schematic diagram of the overall experimental system structure; **(B)** Schematic diagram of the system hardware setup; **(C)** Illustration of patient operation during actual VR training session: A patient with right-sided hemiplegia wore a HMD and a Myo armband on the left forearm. By using the sEMG signal from the left wrist movement, the patient controlled the virtual ankle on their right side.

As depicted in Figure 2 the virtual avatar was placed on a yoga mat in a sitting position with spread legs, with a VR head-mounted display (HMD) providing the viewpoint in a first-person perspective. The patient wore a Myo armband on the unaffected (opposite) side and straightened their arm, performing wrist dorsiflexion repeatedly, constantly and smoothly, affecting the contraction and diastole strength of all forearm muscles. In order to substantially increase the recognition impact of sEMG signal, EMG patch was placed in the portions with high volume for each muscle group and minimal interference from surrounding muscle groups. sEMG signals for the individual's forearm were obtained with an sEMG armband to control the virtual avatar's ankle. The avatar's feet were fastened with strings attached to treasure chests containing gold bars. Each patient performed wrist dorsiflexion to pull these treasure chests as close as possible towards his/her bodies. In the VR scenario, the equivalent virtual ankle dorsiflexion was mapped simultaneously.

### 2.4 KVIQ-10 assessment procedures

The KVIQ-10 questionnaire assesses both visual (V) and kinesthetic (K) subscales of motor imagery. It includes 5 movements per subscale (totally ten items) reflecting gestures with various body parts, i.e., forward shoulder flexion, thumb finger opposition, forward trunk flexion, hip abduction and foot tapping. The test was performed with the patient sitting on a chair with a backrest and the examiner sitting in front of them. Each item was assessed as follows: (1) the patient assumed the starting position per the examiner's demonstration; (2) the examiner for demonstration performed the movement, which was executed by the subject physically once (in case of inability to execute the movement physically with the affected limb, the unaffected limb was employed); (3) the patient was requested to resume the starting position and to imagine carrying out the movement they just executed; (4) the examiner asked the patient to rate the visual image for clarity or the intensity of the sensations associated with the imagined movement on a 5-point scale (Table 2). The patients rated the imagery with operational definitions for various categories (e.g., 5, image as clear as seeing) and the resulting numbers were further analyzed. For items that involve limb movement, both sides were assessed: for items #1 and #2, the opposite side was assessed following item #2 testing; for #4 and #5, the opposite side was assessed after item #5 testing (Table 3). Visual imagery was examined first, followed by kinesthetic imagery (Tables 2, 3).

**Table 3:** KVIQ10 items.

**Visual**	**Kinesthetic**
1Vl	1Kl
2Vr^a^	2Kr^a^
3V	3K
4Vr	4Kr
5Vl^b^	5Kl^b^

l, left limb; r, right limb.

^a^For items #1 and #2; the opposite side was assessed after #2 testing.

^b^For items #4 and #5; the opposite side was assessed after #5 testing.

### 2.5 Self-report measures

In the experiment, participants were asked to fill out some questionnaires. After the training, the subjects were asked about the motor imagery vividness using a visual analogue scale (VAS: 0, no sensation; 10, sensations as intense as if really carrying out the movement) and motor imagery effort (0, no effort; 10, extreme effort). The Likert scale (LS) was employed to assess kinesthetic illusion, sense of body ownership, and sense of agency and motor control (38). For kinesthetic illusion, “I feel like my foot is moving” was utilized as statement; for the sense of body ownership, “The image of the foot feels like a part of my body” was used; for the sense of agency and motor control, “It feels like I could control the virtual ankle as if it is my own ankle” was employed. The participants selected a level of agreement/disagreement based on a 7-point Likert scale for respective statements, with 1 and 3 indicating strong disagreement and agreement, respectively. The raw task load index (Raw TLX) (39), a simplified version of the NASA Task Load Index (40), was employed to assess workload. The intrinsic motivation inventor (IMI) is an evaluation instrument assessing the subjective experience of participants in relation to a given activity in laboratory assays (41). After the IMI assessment, an open interview was conducted regarding 5 and 6 in the IMI. Pre- and post-testing, the simulator sickness questionnaire (SSQ) was employed to measure the symptoms of simulator sickness (42). In addition, participants conducted a semi-structured interview 1 week after the experiment, where they were asked: “Did you often recall the VR scene days ago during this week and try to perform it?”.

THE POST-EXPERIMENTAL INTRINSIC MOTIVATION INVENTORY (IMI)

For each statement below, please indicate how true it is for you, based on the following scale:

1 2 3 4 5 6 7

not at all true somewhat true very true

Q1: I enjoyed performing this activity very much. ()

Q2: I think I am pretty good at this activity. ()

Q3: I put a lot of effort into this. ()

Q4: I did not feel nervous at all while doing this. ()

Q5: I believe I had some choice about doing this activity. ()

Q6: I believe this activity could be of some value to me. ()

**Text 1. The Intrinsic Motivation Inventory (IMI) for all participants**. After the IMI assessment, an open interview was conducted regarding Q5 and Q6; subjects may be asked why they thought the system was valuable for them and why they would like to use the system again if they selected more than 4 as a score in the IMI assessment.

### 2.6 Statistical analyses

Statistical analyses were performed using SPSS (version 27; IBM, Armonk, NY, USA). The normality of data distribution was assessed using the Shapiro-Wilk test with a significance threshold of α = 0.05. Based on the results, appropriate statistical tests were selected: independent *t-*tests for normally distributed independent samples, Mann-Whitney U tests for comparisons involving one normally distributed group and one non-normally distributed group or two non-normally distributed independent samples, and Wilcoxon signed-rank tests for non-normally distributed correlated samples.

To control for type I error inflation due to multiple comparisons, the Bonferroni correction was applied within logically related families of tests. The significance threshold (α) was adjusted by dividing 0.05 by the number of comparisons within each family (i.e., α_adjusted = 0.05/*k*, where *k* is the number of tests in the family). Corrected *p-*values are reported as P_corrected.

Effect sizes were calculated to quantify the magnitude of observed effects. For independent *t-*tests, Cohen's *d* was computed as: *d* = (*M*1 – *M*_2_)/SD_pooled, where SD_pooled is the pooled standard deviation. For Mann-Whitney U and Wilcoxon signed-rank tests, the r effect size was calculated as: *r* = *Z*/√*N*, where *N* is the total number of observations (for independent samples) or the number of paired observations (for correlated samples).

Effect sizes were interpreted according to Cohen's conventions: for Cohen's *d*, 0.20, 0.50, and 0.80 represent small, medium, and large effects, respectively; for *r*, 0.10, 0.30, and 0.50 represent small, medium, and large effects, respectively. Effect sizes below the small effect threshold were considered negligible (43).

Data are reported as mean ± standard error of the mean (SEM) as appropriate. Statistical significance was set at *P* < 0.05 prior to correction, and the corrected significance threshold is reported for each family of tests. All tests were two-tailed.

## 3 Result

The assumption of normality was tested for all variables using the Shapiro-Wilk test. Detailed results of these tests are presented in Supplementary material 1. Statistical methods were chosen accordingly, as detailed in the Methods section.

### 3.1 Motor imagery ability (KVIQ)

**Figure 3:**
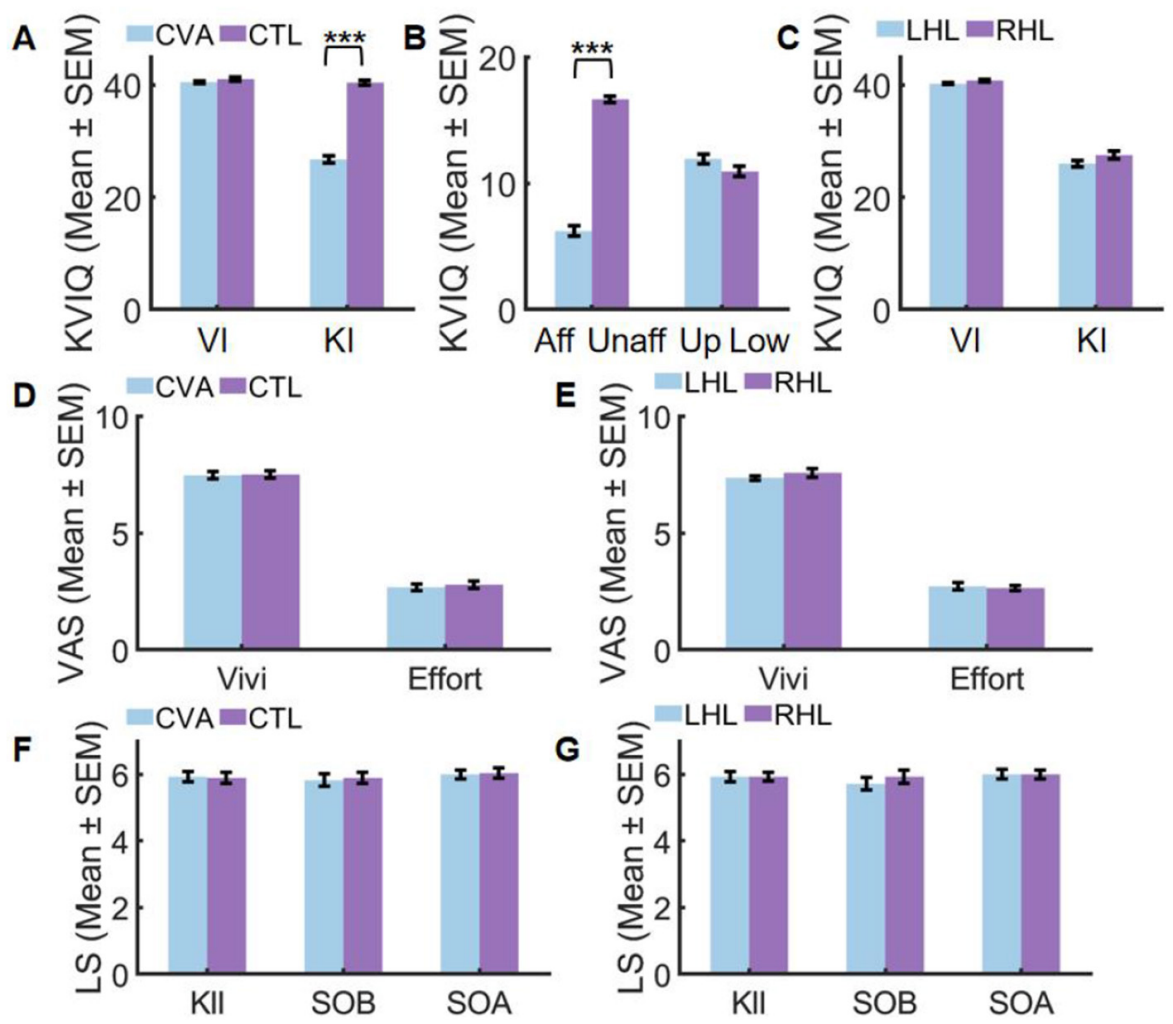
Performance before **(A–C)** and after VR training **(D–G)**. Aff, affected side; CTL, control group; CVA, cerebrovascular accident group; Effort, imagery effort; KI, Kinesthetic Imagery; KIl, Kinesthetic Illusion; LHL, left hemispheric lesions; Low, lower limb; RHL, right hemispheric lesions; SEM, standard error of mean; SOA, Sense of Agency and Motor Control; SOB, Sense of Body Ownership; Unaff, unaffected side; Up, upper limb; VI, visual Imagery; Vivi, imagery vividness (***P_adj_ < 0.001).

According to Figure 3A, CVA demonstrated comparable visual imagery ability (*U* = 482.500, *Z* = 1.529, *P*_adj_ = 0.252, *r* = 0.20) to CTL, whereas their kinesthetic imagery ability was significantly lower (*U* = 784.000, *Z* = 6.450, *P*_adj_ < 0.001, *r* = 0.86).

The kinesthetic imagery ability of patients, as depicted in Figure 3B, revealed a significant difference between the affected and unaffected sides after correction (*t* = 20.849, *P*_adj_ < 0.001, Cohen's *d* = 3.94), whereas no significant difference was observed between the upper and lower limbs (*U* = 292.000, *Z* = 1.673, *P*_adj_ = 0.188, *r* = 0.22). Further details can be found in Supplementary 2 Table 1.

Conversely, Figure 3C illustrates that there was no significant difference in visual imagery ability (*U* = 123.500, *Z* = 1.237, *P*_adj_=0.492, *r* = 0.23) and kinesthetic imagery ability (*U* = 125.500, *Z* = 1.270, *P*_adj_ = 0.420, *r* = 0.24) between right hemispheric lesions (RHL) and left hemispheric lesions (LHL). Further details can be found in Supplementary 2 Table 1.

### 3.3 Subjective imagery experience

After applying Bonferroni correction for multiple comparisons (adjusted α = 0.025), subjective imagery experience showed no significant differences between groups. In the CVA vs. CTL comparison (Figure 3D), motor imagery vividness demonstrated non-significant results (*U* = 407.000, *Z* = 0.264, *P*_adj_ = 1.000, *r* = 0.04) while motor imagery effort similarly showed no significant difference (*U* = 411.000, *Z* = 0.336, *P*_adj_ = 1.000, *r* = 0.04), both exhibiting negligible effect sizes (*r* < 0.05). For the LHL vs. RHL comparison (Figure 3E), motor imagery vividness was non-significant (*U* = 110.500, *Z* = 0.626, *P*_adj_ = 1.000, *r* = 0.12) and motor imagery effort showed comparable non-significant results (*U* = 90.500, *Z* = −0.378, *P*_adj_ = 1.000, *r* = −0.07), with effect sizes ranging from negligible to small (*r* ≤ 0.12). Complete statistical details are available in Supplementary 2 Table 2.

### 3.4 Sense of embodiment (SOE)

After Bonferroni correction for multiple comparisons, no significant group differences emerged in SOE measures. For the CVA vs. CTL comparison (Figure 3F), non-significant results were found across all components including kinesthetic illusion (*U* = 386.500, *Z* = −0.096, *P*_adj_=1.000, *r* = −0.01), sense of body ownership (*U* = 406.500, *Z* = 0.250, *P*_adj_ = 1.000, *r* = 0.03), and sense of agency and motor control (*U* = 402.500, *Z* = 0.183, *P*_adj_ = 1.000, *r* = 0.02), all exhibiting negligible effect sizes (*r* < 0.05). Similarly, in the LHL vs. RHL comparison (Figure 3G), non-significant outcomes were observed for kinesthetic illusion (*U* = 98.500, *Z* = 0.025, *P*_adj_=1.000, *r* = 0.00), sense of body ownership (*U* = 110.500, *Z* = 0.603, *P*_adj_=1.000, *r* = 0.11), and sense of agency and motor control (*U* = 98.000, *Z* = 0.000, *P*_adj_=1.000, *r* = 0.00), with effect sizes ranging from negligible to small (*r* ≤ 0.11). Complete statistical details are documented in Supplementary 2 Table 3.

### 3.5 Intrinsic motivation inventor (IMI)

**Figure 4:**
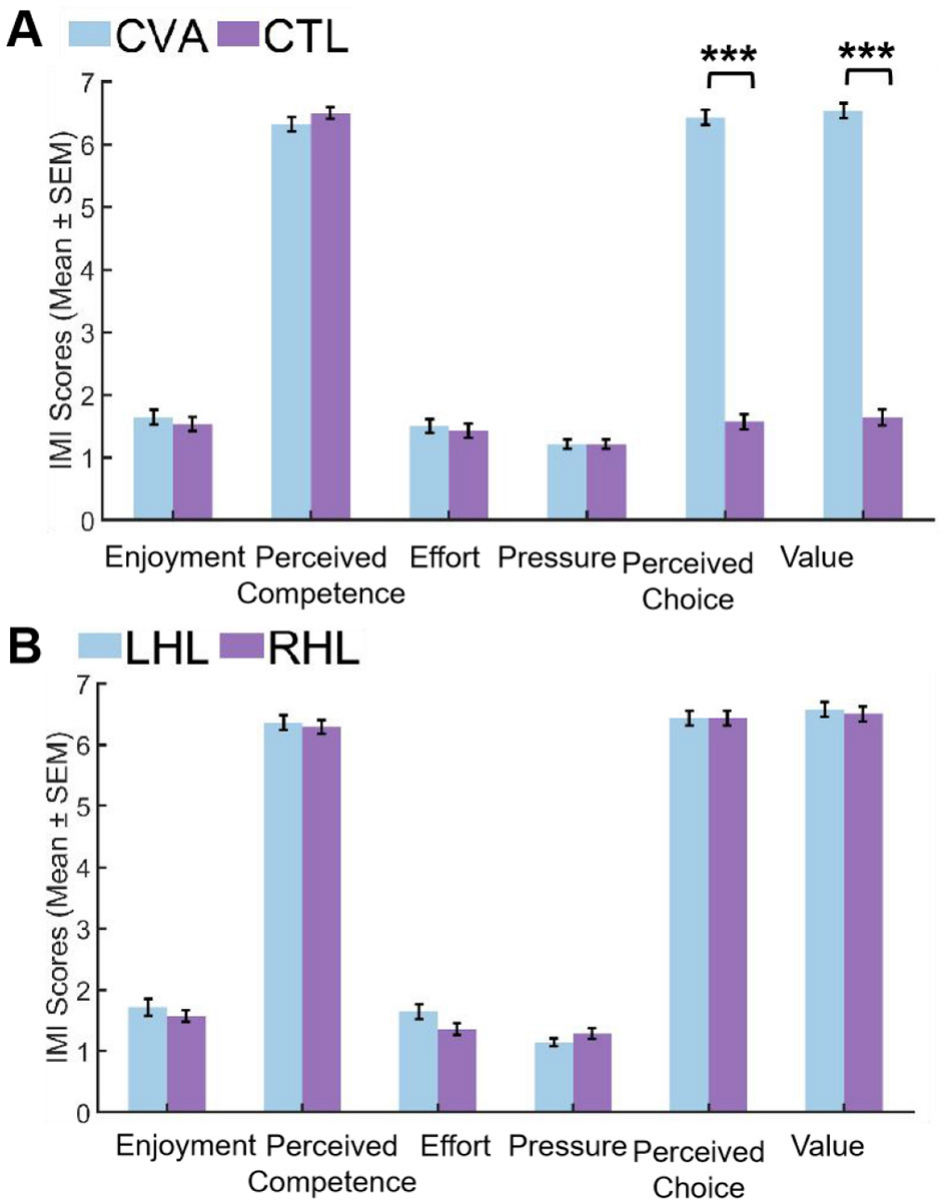
Comparison of IMI scores between groups. **(A)** CVA group vs. CTL group. **(B)** LHL group vs. RHL group. CTL, control group; CVA, cerebrovascular accident group; LHL, left hemispheric lesions; RHL, right hemispheric lesions; SEM, standard error of mean (***P_adj_ < 0.001).

Following Bonferroni correction for multiple comparisons, IMI subscale analysis revealed distinct patterns between groups. In the CVA vs. CTL comparison (Figure 4A), CVA demonstrated significantly higher scores with extremely large effect sizes in both perceived choice (*U* = 0.000, *Z* = −6.593, *P*_adj_ < 0.001, *r* = −0.88) and value (*U* = 0.000, *Z* = –6.606, *P*_adj_ < 0.001, *r* = −0.88). Conversely, no significant differences emerged across enjoyment (*U* = 358.000, *Z* = −0.628, *P*_adj_ = 1.000, *r* = −0.08), perceived competence (*U* = 448.000, *Z* = 1.044, *P*_adj_ = 1.000, *r* = 0.14), effort (*U* = 365.000, *Z* = −0.510, *P*_adj_ = 1.000, *r* = −0.07), and pressure (*U* = 392.000, *Z* = 0.000, *P*_adj_ = 1.000, *r* = 0.00), all showing negligible effect sizes (*r* ≤ 0.14). For the LHL vs. RHL comparison (Figure 4B), all subscales showed non-significant results after correction including enjoyment (*U* = 90.000, *Z* = −0.412, *P*_adj_=1.000, *r* = −0.08), perceived competence (*U* = 91.500, *Z* = −0.337, *P*_adj_=1.000, *r* = −0.06), effort (*U* = 74.500, *Z* = –1.232, *P*_adj_=1.000, *r* = −0.23), pressure (*U* = 112.000, *Z* = 0.905, *P*_adj_=1.000, *r* = 0.17), perceived choice (*U* = 98.000, *Z* = 0.000, *P*_adj_=1.000, *r* = 0.00), and value (*U* = 91.500, *Z* = −0.346, *P*_adj_=1.000, *r* = −0.07), with effect sizes ranging from negligible to small (*r* ≤ 0.23). Complete statistical details are available in Supplementary 2 Tables 4 and 5.

During the semi-structured interview conducted 1 week later, it was discovered that 20 out of 28 patients (71%) frequently recalled scenes from the VR experience in the following week. Moreover, these patients actively employed their imagination to practice ankle movements. In contrast, none of the participants in CTL reported engaging in comparable practices. These findings suggest that the VR experience had a significant influence on the patients' ability to recall and utilize motor imagery techniques even after the intervention had concluded.

### 3.6 Simulator sickness questionnaire (SSQ)

After Bonferroni correction for multiple comparisons, no significant differences in SSQ scores were found between pre- and post-VR exposure for either group. CVA Participants: Oculomotor (*Z* = 2.066, *P*_adj_ = 0.156, *r* = 0.39),Total Severity (*Z* = 2.671, *P*_adj_ = 0.032, *r* = 0.50), Nausea (*Z* = 1.633, *P*_adj_ = 0.408, *r* = 0.31), Disorientation (*Z* = 1.841, *P*_adj_ = 0.264, *r* = 0.35), CTL Participants:Oculomotor (*Z* = 2.111, *P*_adj_ = 0.140, *r* = 0.40), Total Severity (*Z* = 1.897, *P*_adj_ = 0.232, *r* = 0.36), Nausea (*Z* = 0.577, *P*_adj_ = 1.000, *r* = 0.11), Disorientation (*Z* = 1.000, *P*_adj_ = 1.000, *r* = 0.19). Complete statistical details are available in Supplementary 2 Tables 6 and 7.

### 3.7 Raw TLX

**Figure 5:**
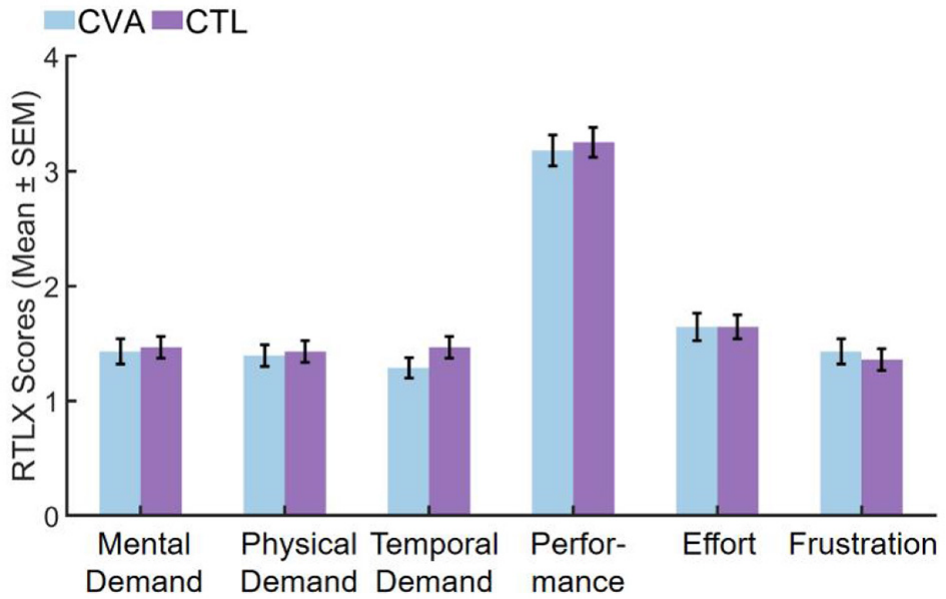
Comparison of RTLX subscales scores in the CVA and CTL groups. CTL, control group; CVA, cerebrovascular accident group; SEM, standard error of the mean.

After Bonferroni correction for multiple comparisons with an adjusted α threshold of 0.0083, no significant differences emerged in any RTLX subscales between CVA and CTL participants. All subscales demonstrated non-significant results including mental demand (*U* = 413.500, *Z* = 0.408, *P*_adj_ = 1.000, *r* = 0.05), physical demand (*U* = 406.000, *Z* = 0.269, *P*_adj_ = 1.000, *r* = 0.04), temporal demand (*U* = 462.000, *Z* = 1.368, *P*_adj_ = 1.000, *r* = 0.18), performance (*U* = 412.500, *Z* = 0.366, *P*_adj_ = 1.000, *r* = 0.05), effort (*U* = 397.000, *Z* = 0.093, *P*_adj_ = 1.000, *r* = 0.01), and frustration (*U* = 373.000, *Z* = −0.369, *P*_adj_=1.000, *r* = −0.05), with temporal demand showing the largest effect size (*r* = 0.18) though still within the small effect range. All effect sizes remained negligible to small (*r* ≤ 0.18), indicating minimal between-group differences in perceived task load. Complete results are visualized in Figure 5 and detailed in Supplementary 2 Table 8.

## 4 Discussion

### 4.1 Motor imagery ability upon a unilateral cerebral lesion

In this study, stroke patients with relatively low scores on the KVIQ-10 were selected. The major finding was that stroke patients presented worse MI ability compared with the control group, particularly in kinesthetic imagery (*r* = 0.86). Regardless of the side of the hemispheric lesion, stroke cases had higher visual than kinesthetic imagery capacity, consistent with previous reports (44).

Another key finding was the asymmetry in MI ability between the affected and unaffected sides in patients, with kinesthetic imagery being significantly impaired on the affected side (Cohen's *d* = 3.94) (44, 45). Since the KVIQ-10 includes both upper and lower limb tasks, we further compared imagery performance across limbs. Although no significant difference was found between upper and lower limbs on the affected side, the observed effect size was small (*r* = 0.22), suggesting that any potential difference may be limited. Similarly, no significant differences were observed in either visual (*r* = 0.23) or kinesthetic imagery (*r* = 0.24) between patients with right and left hemispheric lesions. The small effect sizes indicate that lesion laterality may not substantially influence motor imagery ability in this cohort.

These findings indicate that stroke patients with impaired MI abilities, identified via KVIQ-10, exhibit pronounced kinesthetic imagery deficits on the affected side, with no strong evidence for differential impairment between limbs or based on lesion side.

### 4.2 Effects on the SOE

In the present study, no statistically significant differences were found between stroke patients and healthy controls on any dimension of the SOE—including kinesthetic illusion, body ownership, and agency—under VR conditions. All comparisons yielded negligible effect sizes (*r* < 0.05 for CVA vs. CTL; *r* ≤ 0.11 for LHL vs. RHL), suggesting that the lack of significant differences likely reflects genuinely small between-group disparities rather than insufficient statistical power.

However, Borrego et al. (46) found that compared with the healthy group, lower body-ownership and self-location scores were found in stroke cases. These authors thought that post-stroke cognitive disorders following stroke may challenge the incarnation of virtual avatars (47).

Indeed, our design of the virtual avatar differs significantly from that of Borrego et al., which could be a major factor contributing to the differences obtained in experimental results. Embodied self-representations in a virtual setting constitute an anchor for visuomotor tasks and their morphologies have behavioral consequences. Previous studies have found that the modification of motor parameters in movements during action observation and motor imagery, including force requirements, muscle contraction characteristics, limb movement trajectories, and visual stimuli of object mass can modulate corticospinal excitability or the primary motor cortex area (M1) (48–50). In this study, the entire lower limb without clothing was boldly presented, and the dynamic variations in lower limb muscle movements were realistically depicted. Additionally, ankle movement was designed as a transitive action, where ankle dorsiflexion resulted in the pulling of a box of gold. We believed that the dynamic contraction of lower limb muscles and the movement of the box of gold in the VR scene both generated strong visual stimuli, leading to intense illusions in the participants. In this study, one healthy participant reported feeling a sensation of warmth in his leg when observing the muscle contractions in the VR. However, in Borrego et al.'s (35) VR scene, the participant's lower limb was partially covered with trousers and shoes, concealing any muscle or skin-associated cues related to the applied force. This could be a reason for the weaker sense of bodily illusion experienced in the latter study. Observation, imagination, imitation, learning, and visual feedback are core mechanisms of virtual reality therapy, with the mirror neuron system playing a crucial role; therefore, investigating the subjective mechanisms underlying exposure to virtual reality in stroke patients can have an impact on their experience and, ultimately, improve patient outcomes following neurorehabilitation interventions.

The limited body motion-tracking obtained with the Kinect v2 (51) and insufficient avatar mobility may prevent the reproduction of some pathological motor patterns by their virtual selves in stroke patients, which may reduce their identification of the avatar as their own body. This is likely why stroke cases reported control over the avatar movements but not the ownership or self-location.

In contrast, in the current system, we accurately mapped real-time muscle movements of the unaffected wrist to the virtual ankle movement, inducing a strong sense of agency and enhancing bodily illusions (52). Although the actual physical limb movements in this study differed from those of virtual limbs, participants were still able to experience a strong SOE. These results corroborated previous findings. For example, Tsakiris et al. (53) found that during active movement of one finger, proprioceptive drifts are not localized to the given finger but may spread to the whole hand. Similarly, Merians et al. (52) discovered that viewing a virtual hand corresponding to the individual's affected side and moving it via action by the patient's unaffected hand may selectively facilitate motor areas in the affected hemisphere. The current study extends prior findings by demonstrating that bodily illusions can be induced through the control of virtual limbs using non-homologous limb movements. This may be because somatotopy in the motor cortex is integrated and overlapping (54, 55). So integration of different body parts into a coherent and unified awareness of the body's motor sense of agency is possible.

The results of the present study supported that the SOE is experienced in a similar fashion in stroke patients and the healthy control group. This suggested the primary mechanisms modulating these phenomena may be maintained post-stroke and might imply that VR interventions are effective in stroke cases.

### 4.3 Effects on subjective imagery experience

Under VR conditions, stroke patients with MI impairment reported levels of vividness and effort comparable to age-matched healthy controls. Critically, all group comparisons related to subjective imagery experience yielded non-significant results with negligible to small effect sizes (*r* ≤ 0.12), indicating that the absence of significant differences reflects a genuine similarity in subjective experience between groups rather than a lack of statistical power.

MI can be subdivided into visual (VMI) and kinesthetic (KMI) motor imagery types (56). KMI enhances M1 excitability in comparison with VMI (30), and the KMI ability correlates with M1 excitability (31). However, KMI is more difficult to imagine and less accurate than VMI (57), and methods to improve KMI are limited (58).

One way to improve MI is action observation (59, 60). Previous studies have demonstrated VR-based action observation enhances MI via both visual data and embodiment (18, 61, 62).

Especially in a study of Kishor et al. individuals had enhanced kinesthetic MI-triggered ERD response with VR in comparison with the condition without visual presentation. When participants observed a motor task in an immersive VR setting, the strong sense of body ownership generated can potentially improve the kinesthetic imagery of the body parts contributing to the motor task (62). The current results were consistent with previous studies, even in patients with noticeable impairment in motor imagery ability.

Additionally, most patients reported frequently recalling the video scenes and spontaneously practicing the movements during a 1-week follow-up, i.e., through short-term VR training, patients can establish stable and enduring motor memory. The memory effect may persist for a period of time after the training and positively impacts the patients' motor imagery abilities. Even after the training has ended, patients may still retain the memory of the training content and actively engage in related motor imagery exercises in their daily lives.

There was no difference in subjective imagery experience observed between patients with left or right brain damage. A patient with right brain damage causing hemispatial neglect expressed a strong sense of embodiment and enjoyed the experience. These results implied that the design of our VR training system was feasible for most stroke patients even with some cognitive impairment in the early stage.

Furthermore, this study involved patients actively controlling the movements of their virtual avatars. The sense of agency and motor control exertion were also important in enhancing motor imagery. In a study of MI-BCI combined with virtual reality, many subjects expressed that controlling an avatar embodying them facilitates MI (63, 64). The current patients also expressed similar experiences.

In summary, in this study, the strong sense of body ownership illusions, visual motion illusions, and sense of agency collectively contributed to the improvement of KMI performance in individuals with impaired MI abilities.

### 4.4 Effects on motivation

The IMI represents the commonest tool for evaluating motivation in various contexts (65). However, it has limited sensitivity in detecting the effect on motivation of specific factors of the assigned task, likely because it assessed motivation in general aspects in lieu of specific ones (66). Therefore, we conducted open-ended interviews immediately after the IMI assessment and again 1 week later to further examine the specific factors influencing motivation during the task and the internalization process following the completion of the treatment.

IMI results revealed a distinct motivational profile between stroke patients (CVA) and healthy controls (CTL). Patients reported significantly higher scores in perceived choice and perceived value, with extremely large effect sizes (*r* = |0.88|), indicating a strong, clinically meaningful difference. In contrast, no significant differences were found between groups on the subscales of enjoyment, perceived competence, effort, and pressure. Critically, the effect sizes for these comparisons were negligible (*r* ≤ 0.14), supporting the interpretation that patients and controls were genuinely similar in these aspects of motivational experience. Furthermore, no significant differences were observed on any IMI subscale between patients with left and right hemispheric lesions, with all comparisons showing small to negligible effect sizes (*r* ≤ 0.23).

Consistent with Self Determination Theory (SDT), motivation can be intrinsic—driven by interest and enjoyment—or extrinsic—reliant on external incentives (67). Our system was intentionally designed without gamified elements, and the virtual scenes were kept simple. Notably, non-impaired individuals did not report intrinsic interest or perceive value in the system, as they had no motor deficits to address. In contrast, although patients also did not find the system inherently enjoyable, they consistently regarded it as valuable and expressed willingness to reuse it.

It is important to consider the context in which the tasks were performed. Patients were in a recovery phase, often still largely confined to hospital settings, which may amplify the perceived value of any intervention that could potentially aid rehabilitation. This situational factor could contribute to the higher scores in perceived choice and value among patients compared to controls, as the opportunity to engage in a task that might improve motor function naturally holds greater relevance and meaning for them.

Nevertheless, post-assessment interviews revealed that patients attributed the value they placed on the system specifically to the vivid bodily illusions and improvements in motor imagery it facilitated. Although these elements did not directly translate into functional gains, they were perceived as being linked to motor recovery. Moreover, the majority of patients spontaneously recalled and used the VR experience for self-training 1 week later—a phenomenon not reported in the control group.

These observations suggest that the system supported the satisfaction of key psychological needs such as autonomy and competence, for instance, by enabling control of the virtual foot via wrist movement, thereby enhancing patients' confidence and agency. This fulfillment of basic psychological needs appears to have facilitated a form of self-regulation, enabling the internalization of extrinsic motivation even in the absence of immediate enjoyment or interest (68).

Thus, while the clinical context may heighten the perceived value of rehabilitation activities, our findings indicate that the specific attributes of the VR intervention—particularly its support of bodily illusion and motor imagery—also played a critical role in fostering motivation. This underscores the importance of designing rehabilitation technologies that not only address physical needs but also incorporate elements that enhance psychological engagement, thereby promoting sustained motivation and participation throughout the recovery process.

### 4.5 Adverse reactions

Immersive VR-based tools can potentially induce adverse effects such as motion sickness, dizziness, and visual disturbances, raising concerns about their applicability in neuromotor rehabilitation for brain-injured patients (69). It is therefore noteworthy that in this study, no statistically significant differences in SSQ scores were observed before vs. after VR exposure in either stroke patients or healthy controls. However, pre-post comparisons yielded effect sizes ranging from small to moderate (*r* ≤ 0.50). In particular, the total severity score among stroke patients showed a moderate effect (*r* = 0.50), and a small to moderate effect was observed in healthy controls (*r* = 0.36). Although these changes were not statistically significant, the magnitude of change—especially in patients—warrants careful interpretation.

Similarly, no significant between-group differences were detected across any dimension of task load (Raw TLX), with all effect sizes being negligible to small (*r* ≤ 0.18). The consistent lack of statistical significance across outcomes, combined with mostly small effects on task load, suggests that the VR system did not impose a substantial perceived burden during use. Nevertheless, the moderate effect size for total severity in patients indicates that some individuals may have experienced non-negligible symptoms, even if group-level significance was not reached.

The generally low adverse effect profile may be attributed to the study's seated design, which required all participants to remain in bed during training. This stable, supported positioning likely reduced vestibular conflict and physical strain, contributing to the overall tolerability of the system. Nonetheless, the observed moderate effect in symptom severity among patients highlights the importance of individual monitoring and further investigation into patient-specific factors that may influence VR tolerance.

## 5 Limitation

In this study, subjective measures were employed as assessment methods, and objective physiological indicators were not utilized. However, we believe that subjective evaluations remain significant for our VR feedback system and its impact on patients. Neurorehabilitation must consider the multiple aspects of a patient by comprehensively analyzing actual and possible cognitive, behavioral, emotional and physical skills, while enhancing awareness and understanding of the new self of the patient in question. Exclusive application of objective functional parameters by the rehabilitator generally overlooks the values and goals of the disabled individual. Indeed, each patient has unique rhythm, life history and personality. Therefore, it is critical to deepen the evaluation by including subjective indexes that more accurately reflect the individual's perspective, to build tailored neurorehabilitation strategies in lieu of standardized ones.

Additionally, the participant cohort consisted exclusively of patients in the early post-stroke stage. This focus was a deliberate choice for this initial validation, as our primary goal is to provide a rehabilitation pathway for severe, bedridden patients with motor imagery deficits who have very limited active training options at this critical stage. Consequently, the findings may not be generalizable to chronic stroke patients, who often have access to a wider array of active rehabilitation paradigms. Future work will explore the efficacy and adaptation of this system for patients in the chronic phase of stroke recovery.

## 6 Conclusion

In conclusion, the sense of embodiment in VR, comprising self-location, agency, and body ownership, plays a crucial role in enhancing motor imagery ability. By creating a strong sense of embodiment, VR optimizes the effectiveness of motor rehabilitation interventions and enhances the overall rehabilitation experience of stroke patients. When designing virtual reality interventions for rehabilitation, it is crucial to have a comprehensive understanding of how various sensory and motor manipulations in virtual reality impact neurological processes. This is the only way to fully harness the potential of virtual reality and maximize its effectiveness in rehabilitation settings.

## Data Availability

The original contributions presented in the study are included in the article/Supplementary material, further inquiries can be directed to the corresponding author/s.
