# Supplementary material for: Vivid illusions and realtime feedback in VR-improved motor imagery and motivation of stroke patients with impaired motor imagery
Source: Front Neurol. 2025 Nov 7;16:1629587. doi: 10.3389/fneur.2025.1629587 (PMC12637228; doi:10.3389/fneur.2025.1629587)
Supplement: Supplementary file 1 [file Table_1.docx]

DATA DISTRIBUTION CHARACTERISTICS

Comprehensive assessment of variable distributions revealed heterogeneous normality profiles across measures. The Shapiro-Wilk test demonstrated were followed：

***KVIQ***

|  | W | p | Conclusions |
| --- | --- | --- | --- |
| CVA VI | 0.91176 | 0.02181 | Non-normal distribution |
| CTL VI | 0.9697 | 0.57244 | Normal distribution |
| CVA KI | 0.92172 | 0.03827 | Non-normal distribution |
| CTL KI | 0.92299 | 0.04114 | Non-normal distribution |
| Up KI | 0.90933 | 0.01905 | Non-normal distribution |
| Low KI | 0.66548 | <0.0001 | Non-normal distribution |
| Aff KI | 0.8904 | 0.06801 | normal distribution |
| Unaff KI | 0.92727 | 0.05265 | Normal distribution |
| LHL VI | 0.8226 | 0.00323 | Non-normal distribution |
| RHL VI | 0.91094 | 0.12049 | Normal distribution |
| LHL KI | 0.90006 | 0.05759 | Normal distribution |
| RHL KI | 0.88302 | 0.04325 | Non-normal distribution |

Aff: affected side;CTL: control group; CVA: cerebrovascular accident group;KI: Kinesthetic Imagery;LHL: left hemispheric lesions ;Low: lower limb; RHL:right hemispheric lesions;Unaff: unaffected side; Up: upper limb; VI: visual Imagery.

***Subjective Imagery Experience***

|  | W | p | Conclusions |
| --- | --- | --- | --- |
| CVA Vivi | 0.84837 | 8.66845E-4 | Non-normal distribution |
| CTL Vivi | 0.87609 | 0.0033 | Non-normal distribution |
| CVA effort | 0.84206 | 6.4917E-4 | Non-normal distribution |
| CTL effort | 0.78722 | <0.0001 | Non-normal distribution |
| LHL Vivi | 0.63034 | <0.0001 | Non-normal distribution |
| RHL Vivi | 0.88512 | 0.08368 | Normal distribution |
| LHL effort | 0.86456 | 0.0281 | Non-normal distribution |
| RHL effort | 0.77231 | 0.00326 | Non-normal distribution |

CTL:control group; CVA:cerebrovascular accident group; Effort: imagery effort; LHL: left hemispheric lesions ;RHL:right hemispheric lesions; Vivi: imagery vividness.

***Sense Of Embodiment (SOE)***

|  | W | p | Conclusions |
| --- | --- | --- | --- |
| CVA KII | 0.81018 | 1.62252E-4 | Non-normal distribution |
| CTL KII | 0.85534 | 0.0012 | Non-normal distribution |
| CVA SOB | 0.84974 | 9.23484E-4 | Non-normal distribution |
| CTL SOB | 0.83935 | 5.7413E-4 | Non-normal distribution |
| CVA SOA | 0.86046 | 0.00153 | Non-normal distribution |
| CTL SOA | 0.80584 | 1.35511E-4 | Non-normal distribution |
| LHL KII | 0.79921 | 0.00359 | Non-normal distribution |
| RHL KII | 0.82037 | 0.01202 | Non-normal distribution |
| LHL SOB | 0.84248 | 0.01362 | Non-normal distribution |
| RHL SOB | 0.87925 | 0.06966 | Normal distribution |
| LHL SOA | 0.80552 | 0.00433 | Non-normal distribution |
| RHLSOA | 0.87764 | 0.06625 | Normal distribution |

CTL:control group; CVA:cerebrovascular accident group; KIl, Kinesthetic Illusion; LHL: left hemispheric lesions ;RHL:right hemispheric lesions;SOA, Sense of Agency and Motor Control;SOB, Sense of Body Ownership.

***Intrinsic Motivation Inventor (IMI)***

|  | W | p | Conclusions |
| --- | --- | --- | --- |
| CVA interest/enjoyment | 0.75762 | <0.0001 | Non-normal distribution |
| CVA perceived competence | 0.75817 | <0.0001 | Non-normal distribution |
| CVA effort | 0.71228 | <0.0001 | Non-normal distribution |
| CVA pressure/tension | 0.50768 | <0.0001 | Non-normal distribution |
| CVA perceived choice | 0.74538 | <0.0001 | Non-normal distribution |
| CVA value | 0.6989 | <0.0001 | Non-normal distribution |
| CTL interest/enjoyment | 0.7199 | <0.0001 | Non-normal distribution |
| CTL perceived competence | 0.63858 | <0.0001 | Non-normal distribution |
| CTL effort | 0.68534 | <0.0001 | Non-normal distribution |
| CTL pressure/tension | 0.50768 | <0.0001 | Non-normal distribution |
| CTL perceived choice | 0.74538 | <0.0001 | Non-normal distribution |
| CTL value | 0.76755 | <0.0001 | Non-normal distribution |

CTL:control group; CVA:cerebrovascular accident group.

***Simulator Sickness Questionnaire(SSQ)***

|  | W | p | Conclusions |
| --- | --- | --- | --- |
| CVA perceived choice | 0.61164 | <0.0001 | Non-normal distribution |
| CVA value | 0.79277 | <0.0001 | Non-normal distribution |
| CVA enjoyment | 0.40807 | <0.0001 | Non-normal distribution |
| CVA perceived competence | 0.61164 | <0.0001 | Non-normal distribution |
| CVA effort | 0.79277 | <0.0001 | Non-normal distribution |
| CVA pressure | 0.40807 | <0.0001 | Non-normal distribution |
| CTL perceived choice | 0.28736 | <0.0001 | Non-normal distribution |
| CTL value | 0.65266 | <0.0001 | Non-normal distribution |
| CTL enjoyment | 0.36062 | <0.0001 | Non-normal distribution |
| CTL perceived competence | 0.28736 | <0.0001 | Non-normal distribution |
| CTL effort | 0.65266 | <0.0001 | Non-normal distribution |
| CTL pressure | 0.36062 | <0.0001 | Non-normal distribution |

CTL:control group; CVA:cerebrovascular accident group.

***Raw TLX***

|  | W | p | Conclusions |
| --- | --- | --- | --- |
| CVA Mental Demand | 0.77896 | <0.0001 | Non-normal distribution |
| CVA Physical Demand | 0.78196 | <0.0001 | Non-normal distribution |
| CVA Temporal Demand | 0.5778 | <0.0001 | Non-normal distribution |
| CVA Performance | 0.75762 | <0.0001 | Non-normal distribution |
| CVA Effort | 0.77352 | <0.0001 | Non-normal distribution |
| CVA Frustration | 0.5778 | <0.0001 | Non-normal distribution |
| CTL Mental Demand | 0.75674 | <0.0001 | Non-normal distribution |
| CTL Physical Demand | 0.67542 | <0.0001 | Non-normal distribution |
| CTL Temporal Demand | 0.66152 | <0.0001 | Non-normal distribution |
| CTL Performance | 0.7805 | <0.0001 | Non-normal distribution |
| CTL Effort | 0.77352 | <0.0001 | Non-normal distribution |
| CTL Frustration | 0.36568 | <0.0001 | Non-normal distribution |

CTL:control group; CVA:cerebrovascular accident group.
