# Supplementary material for: Vivid illusions and realtime feedback in VR-improved motor imagery and motivation of stroke patients with impaired motor imagery
Source: Front Neurol. 2025 Nov 7;16:1629587. doi: 10.3389/fneur.2025.1629587 (PMC12637228; doi:10.3389/fneur.2025.1629587)
Supplement: Supplementary file 2 [file Table_2.docx]

**Supplementary 2** **Table1**

**Motor imagery in stroke patients according to the results of the KVIQ-10 ( means ± SD)**

|  | KVIQ_vis_ | KVIQ_kin_ |
| --- | --- | --- |
| All patients | 40.500(1.106) | 26.750(3.228) |
| Affected side | 17.607(0.737) | 6.214(2.234) |
| Unaffected side | 18.357(0.780) | 16.643(1.420) |
| LHL | 40.786(1.311) | 27.500(3.546) |
| RHL | 40.214(0.802) | 26.000(2.801) |
| Note: Mean(SD); |  |  |

KVIQ-10, Kinesthetic and Visual Imagery Questionnaire; SD,the mean and standard deviation;Vis：visual imagery;Kim:kinestic imagery

**Supplementary 2** **Table 2**

**Motor imagery vividness and effort for each group(mean ±SD)**

|  | Imagery Vividness | Imagery Effort |
| --- | --- | --- |
| CVA | 7.464(0.793) | 2.679(0.723) |
| CTL | 7.500(0.839) | 2.786(0.787) |
| LHL | 7.357(0.497) | 2.714(0.497) |
| RHL | 7.571(1.016) | 2.643(0.633) |
| Note: Mean(SD); |  |  |

CTL: control group; CVA: cerebrovascular accident group；LHL: left hemispheric lesions ;RHL:right hemispheric lesions. SD,the mean and standard deviation.

**Supplementary 2** **Table 3**

**Sense of embodiment for each group(mean±SD)**

|  | Kinesthetic Illusion | Sense of Body Ownership | Sense of Agency |
| --- | --- | --- | --- |
| CVA | 5.929(0.766) | 5.821(0.983) | 6.000(0.720) |
| CTL | 5.893(0.875) | 5.893(0.917) | 6.036(0.838) |
| LHL | 5.929(0.829) | 5.714(0.995) | 6.000(0.785) |
| RHL | 5.929(0.730) | 5.929(0.997) | 6.000(0.679) |
| Note: Mean(SD); |  |  |  |

CTL: control group; CVA: cerebrovascular accident group；LHL: left hemispheric lesions ;RHL:right hemispheric lesions; SD:the mean and standard deviation.

**Supplementary 2 Table 4**

**Differences in each item score of IMI between CVA group and CTL group (mean±SD)**

| Item | CVA | CTL | P-value |
| --- | --- | --- | --- |
| Enjoyment | 1.643(0.622) | 1.536(0.576) | 0.530 |
| Perceived Competence | 6.321(0.612) | 6.500(0.509) | 0.296 |
| Effort | 1.500(0.109) | 1.429(0.573) | 0.610 |
| Pressure | 1.214(0.418) | 1.214(0.418) | 1.000 |
| Perceived Choice | 6.429(0.634) | 1.571(0.634) | *** |
| Value | 6.536(0.637) | 1.643(0.679) | *** |
| Note: Mean(SD); *** p < 0.001 | | | |

CTL: control group; CVA: cerebrovascular accident group;SD:the mean and standard deviation.

**Supplementary 2 Table 5**

**Each item score of IMI in patients with RHL and LHL(mean±SD)**

| Item | LHL | RHL | P-value |
| --- | --- | --- | --- |
| Enjoyment | 1.714(0.726) | 1.571(0.514) | 0.734 |
| Perceived Competence | 6.357(0.633) | 6.286(0.611) | 0.769 |
| Effort | 1.643(0.633) | 1.357(0.497) | 0.285 |
| Pressure | 1.143(0.363) | 1.286(0.469) | 0.541 |
| Perceived Choice | 6.429(0.646) | 6.429(0.646) | 1.000 |
| Value | 6.514(0.646) | 6.500(0.650) | 0.769 |
| Note: Mean(SD); *** p < 0.001 | | | |

LHL: left hemispheric lesions ;RHL:right hemispheric lesions; SD:the mean and standard deviation.

**Supplementary 2** **Table6**

**Comparisons of SSQ ratings before and after performing the task of CVA group(mean±SD)**

| SSQ Subscale | Before | After | P-value |
| --- | --- | --- | --- |
| Nausea | 0.681(2.502) | 2.044(4.757) | 0.102 |
| Oculomotor | 1.895(3.928) | 4.873(8.047) | * |
| Disorientation | 0.000(0.000) | 3.977(11.895) | 0.066 |
| Total Severity | 1.202(2.504) | 4.274(6.957) | ** |
| Note: Mean(SD); * p < 0.05;** p < 0.01  Nausea = Count(Category 1) × 9.54  Oculomotor = Count(Category 2) × 7.58  Disorientation = Count(Category 3) × 13.92  Total Severity = (Count(Category 1, Category 2, Category 3)) × 3.74 | | | |

SSQ includes three categories of symptoms:Category 1:Nausea SymptomsCategory; Category 2:Oculomotor Symptoms;Category 3:Disorientation Symptoms.SD:the mean and standard deviation.

**Supplementary 2 Table 7**

**Comparisons of SSQ ratings before and after performing the task of CTL group (mean±SD)**

| Item | Before | After | P-value |
| --- | --- | --- | --- |
| Nausea | 0.341(1.803) | 0.681(2.502) | 0.564 |
| Oculomotor | 1.895(5.310) | 3.790(7.294) | * |
| Disorientation | 0.000(0.000) | 0.497(2.631) | 0.317 |
| Total Severity | 1.069(2.853) | 2.271(4.115) | 0.058 |
| Note: Mean(SD); * p < 0.05;  Nausea = Count(Category 1) × 9.54  Oculomotor = Count(Category 2) × 7.58  Disorientation = Count(Category 3) × 13.92  Total Severity = (Count(Category 1, Category 2, Category 3)) × 3.74 | | | |

SSQ includes three categories of symptoms:Category 1:Nausea SymptomsCategory; Category 2:Oculomotor Symptoms;Category 3:Disorientation Symptoms.SD:the mean and standard deviation.

**Supplementary 2** **Table 8**

**Differences in each item score of RTLX between CVA group and CTL group (mean±SD)**

| Item | CVA | CTL | P-value |
| --- | --- | --- | --- |
| Mental Demand | 1.429(0.573) | 1.464(0.508) | 0.683 |
| Physical Demand | 1.393(0.497) | 1.429(0.504) | 0.788 |
| Temporal Demand | 1.286(0.460) | 1.464(0.5080) | 0.171 |
| Performance | 3.179(0.723) | 3.250(0.701) | 0.715 |
| Effort | 1.643(0.622) | 1.643(0.559) | 0.926 |
| Frustration | 1.429(0.573) | 1.357(0.488) | 0.712 |
| Note: Mean(SD); | | | |

CTL: control group; CVA: cerebrovascular accident group;SD:the mean and standard deviation.
